# Impact of emollient therapy for preterm infants in the neonatal period on child neurodevelopment in Bangladesh: an observational cohort study

**DOI:** 10.1186/s41043-021-00248-9

**Published:** 2021-05-26

**Authors:** Gary L. Darmstadt, Naila Z. Khan, Summer Rosenstock, Humaira Muslima, Monowara Parveen, Wajeeha Mahmood, A. S. M. Nawshad Uddin Ahmed, M. A. K. Azad Chowdhury, Scott Zeger, Samir K. Saha

**Affiliations:** 1 Prematurity Research Center, Division of Neonatal and Developmental Medicine, Department of Pediatrics, Stanford University School of Medicine, 1701 Page Mill Road, Palo Alto, CA 94304 USA; 2 Clinical Neurosciences Center, Bangladesh Protibondhi Foundation, Dhaka, Bangladesh; 3 Center for American Indian Health, Johns Hopkins Bloomberg School of Public Health, Baltimore, MD USA; 4 Department of Neonatology, Bangladesh Institute of Child Health, Dhaka Shishu Hospital, Dhaka, Bangladesh; 5 Department of Epidemiology, Johns Hopkins Bloomberg School of Public Health, Baltimore, MD USA; 6 Department of Microbiology, Bangladesh Institute of Child Health, Dhaka Shishu Hospital, Dhaka, Bangladesh; 7 Child Health Research Foundation , Dhaka, Bangladesh

**Keywords:** Emollient, Neurodevelopment, Preterm, Follow-up, Newborn, Neonatal

## Abstract

**Background:**

Topical treatment with sunflower seed oil (SSO) or Aquaphor® reduced sepsis and neonatal mortality in hospitalized preterm infants <33 weeks’ gestational age in Bangladesh. We sought to determine whether the emollient treatments improved neurodevelopmental outcomes during early childhood.

**Methods:**

497 infants were randomized to receive SSO, Aquaphor®, or neither through the neonatal period or hospital discharge. 159 infant survivors were enrolled in the longitudinal follow-up study using a validated Rapid Neurodevelopmental Assessment tool and the Bayley Scales of Infant Development II (BSID II) administered at three-monthly intervals for the first year and thereafter at six-monthly intervals. Lowess smoothing was used to display neurodevelopmental status across multiple domains by age and treatment group, and Generalized Estimating Equations (GEE) were used to compare treatment groups across age points.

**Results:**

123 children completed at least one follow-up visit. Lowess graphs suggest that lower proportions of children who received massage with either SSO or Aquaphor® had neurodevelopmental delays than control infants in a composite outcome of disabilities. In GEE analysis, infants receiving SSO showed a significant protective effect on the development of fine motor skills [odds ratio (OR) 0.92, 95% confidence interval (CI) 0.86–0.98, *p*=0.006]. The Psychomotor Development Index (PDI) in the BSID II showed significantly lower disability rates in the Aquaphor group (23.6%) compared to the control (55.2%) (OR 0.21, 95% CI 0.06–0.72, *p*=0.004).

**Conclusions:**

Emollient massage of very preterm, hospitalized newborn infants improved some child neurodevelopmental outcomes over the first 2 years of follow-up. Findings warrant further confirmatory research.

**Trial registration:**

ClinicalTrials.gov (98-04-21-03-2) under weblink https://clinicaltrials.gov/ct2/show/NCT00162747

## Background

Among major causes of under 5-year-old child deaths, global progress has been slowest for reducing deaths due to preterm birth [1]. Preterm birth is now the top worldwide cause of death in children before their fifth birthday.

Prematurity is associated with a variety of adverse outcomes among survivors, including chronic pulmonary, cardiovascular, and metabolic disease; deficits in growth, hearing, vision, cognition, and other domains of development; and behavioral problems, learning difficulties, and poor academic performance [2–9]. Moreover, adverse outcomes can persist through adolescence and into adulthood, limiting educational achievement and adult earning potential [5, 6, 10].

Adverse outcomes among very preterm infants are particularly pronounced in developing countries where there are few resources to mitigate risks. Child disability rates are going up in many emerging economies, due primarily to relatively high rates of disability among preterm infants 28 to 32 weeks gestational age who receive poor quality care during pregnancy, childbirth, and the postpartum period [7, 11]. In our follow-up study of a cohort of preterm infants < 33 weeks’ gestational age in Bangladesh, 68% were found to have one or more disabilities or impairments [12].

Postnatal interventions can potentially improve neurodevelopment for preterm infants worldwide [13–16]. Interventions that begin early, prior to hospital discharge, and continue into early childhood when neurodevelopment is particularly responsive to nurturing interventions [17, 18], might be particularly effective at preventing adverse outcomes [14, 16]. Various strategies to increase weight gain, reduce the length of initial hospital stay, and enhance motor and cognitive development—such as nutritional supplements, physical and sensory stimulation—have been introduced over the past several decades and have met with variable success [13–16, 19].

Massage therapy is practiced in some neonatal intensive care units or intermediate care units in developed and developing countries [19–33], where the most consistently reported benefit among infants receiving massage therapy is greater weight gain for preterm infants [21–25, 29, 34, 35]. Massage may result in a variety of other potential benefits including reduced length of hospital stay and cost savings, increased bone mineralization, and improvement in a variety of functional neurodevelopmental indicators, although with variable results [20, 21, 25, 27–30, 32, 36].

Oil massage of newborn infants—combining massage with applications of a variety of emollients, particularly natural vegetable oils—is a traditional domiciliary practice utilized in many parts of the world, especially countries in the Mediterranean region, South Asia, and sub-Saharan Africa, with a growing literature on use of this approach to promote newborn and child health [29, 33–35, 37–55]. Topical applications of emollients for hospitalized, very preterm infants in low- and middle-income countries (LMICs) have reduced neonatal mortality 27% [relative risk (RR): 0.73, 95% confidence interval (CI): 0.56, 0.94)] and hospital-acquired bloodstream infection by 50% (RR 0.50, 95% CI 0.36, 0.71), and resulted in significant increases in weight (g) [mean difference (MD): 98.04, 95% CI 42.64, 153.45] and weight gain (g/kg/day) (MD 1.57, 95% CI 0.79, 2.36) during the neonatal period compared to control infants [34, 35, 46–48]. Evidence for impact of emollient therapy in preterm infants in LMIC settings is strongest for sunflower seed oil (SSO) [35]. The cost per death averted in Bangladesh was $61 for SSO therapy [56], making this a highly cost-effective intervention. Limited data exists on the impact of oil massage therapy on neurodevelopmental outcomes in infants [28–30, 33, 37, 38, 54].

This study describes the role of topical emollient therapy of preterm infants < 33 weeks gestational age with SSO or Aquaphor® during hospitalization in the neonatal period on neurodevelopmental outcomes during the first two years of childhood compared to infants who received no oil massage in Bangladesh.

## Methods

### Parent trial

We conducted a prospective, randomized, controlled clinical trial of topical emollient therapy among 497 preterm infants in the Special Care Nursery of Dhaka Shishu Hospital from December 1998 to July 2003, as described previously [47–49]. Dhaka Shishu Hospital is the largest pediatric hospital in Bangladesh providing both primary and tertiary care, including comprehensive child developmental and intervention services. The hospital has no delivery facility, and all neonates were out-born.

#### Eligibility and exclusion criteria

Newborn infants <72-hour old and <33-week gestational age who were admitted to the hospital were eligible for enrollment. Gestational age was calculated as an average determined by three measures: Dubowitz and Ballard criteria and on the basis of maternal dates (time from the first day of the last menstrual period) [57–59]. We excluded critically ill babies that the admitting physician, based on clinical judgment, thought would die within 48 h; those who had a major congenital anomaly, hydrops fetalis, or generalized skin disease or a structural defect of >5% body surface area; and those admitted to the hospital for a major surgical procedure.

#### Parent study procedures

After a study physician had confirmed patient eligibility for enrollment, neonates were allocated to strata based on gestational age [(1) <30 weeks or (2) >30 weeks] and randomly assigned by a study nurse to the untreated control group or one of two treatment groups: topical applications of high-linoleate SSO (Omega Nutrition, Bellingham, Washington) or Aquaphor Original Emollient Ointment® (Beiersdorf, Norwalk, Connecticut), as described in detail previously [47]. Briefly, we created lists for enrollment using blocks of six with two assignments per block for all of the three groups. A data manager periodically reviewed the original randomization sequence for each stratum and compared it with the recorded allocations made by the nurses. Study physicians were not involved in the procedures used to randomize infants and did not have access to the randomization lists. However, it was not possible to identify a control emollient with demonstrated safety yet no effect on epidermal barrier function; thus, masking was not done. Nurses applied the emollients when the doctors where not on the ward. Emollients were absorbed within about an hour of application, and routine physician assessments of patients were conducted toward the end of the interval in between applications when there was little to no residual emollient on the skin.

All neonates received a brief lukewarm bath at enrollment to remove potential exogenous substances on the skin. Study nurses washed their hands with soap and water, followed by Dettol disinfectant before emollient applications. Four grams of emollient per kilogram of body weight was applied three times daily for the first 14 days and then twice daily until hospital discharge, to the entire body surface avoiding the scalp, face, and intravascular catheter sites. Nurses were trained and supervised regularly in gentle application techniques to minimize the risk of skin injury and potential for spread of fecal flora. Nurses demonstrated, instructed, and observed families in the SSO and Aquaphor arms in emollient application before discharge from the hospital-based parent trial and encouraged caretakers to continue emollient practices at home.

To reduce contamination potential and oxidative breakdown, SSO was refrigerated and replaced by fresh products every two months. Additionally, fresh containers of SSO were prepared every 2 to 3 days for use at individual patient bedsides; adherence to sterile procedures during dispensing and application was enforced, and weekly cultures of individual patient SSO samples were evaluated for contamination. Aquaphor was supplied by Beiersdorf in small tubes containing enough ointment for one to two applications, eliminating contamination risk. Neonates in the control group received standard skin care for the Special Care Nursery, which did not include topical emollients, massage, or other skin care measures. Background information on demographic and clinical characteristics of patients and their mothers was obtained, including maternal obstetric history, birth history, and medical history for each subject.

### Neurodevelopmental follow-up study design

This was a prospective observational cohort study from January 1999 to July 2005 of the preterm newborn infant survivors from the parent emollient trial at Dhaka Shishu Hospital. Community health workers (CHWs) recorded details of the child’s home address during the hospital stay and counseled the care provider on the need for regular follow-up to assess their child’s neurodevelopment. Subjects were enrolled into the neurodevelopmental follow-up study on discharge and were examined longitudinally for up to two years for neurodevelopmental impairments (NDIs) and disabilities by a multidisciplinary team of child health and development professionals at predetermined intervals at the Child Development Centre (CDC) at Dhaka Shishu Hospital. Strengthening the Reporting of Observational Studies in Epidemiology (STROBE) guidelines for study presentation were followed.

### Subjects

Infants who survived to discharge from the parent trial were enrolled. Because newborn infants come to Dhaka Shishu Hospital from throughout Bangladesh, post-discharge follow-up was not feasible for some families who lived too remotely or too far away. Baseline data on socioeconomic status and family demographics, maternal, and birth history; gestational age; anthropometric measures; and illnesses during hospitalization were collected from the parent study records.

### Follow-up

Neurodevelopmental follow-up started at four weeks postnatal age, continued at three-monthly intervals for the first year, and thereafter occurred at six-monthly intervals. Travel allowance was provided to families whenever required to ensure attendance at follow-up visits. For infants who failed to appear for a visit, a CHW was sent to the home to accompany the child and guardian(s) to the CDC.

Visits included a neurodevelopmental assessment carried out by a child health physician and a psychological assessment performed by a developmental psychologist. These assessments were conducted independently of each other and each took about 30 min to complete. In addition, a full physical examination was performed by the physician, which took about 15 min. The developmental therapist counseled mothers on nutrition and care practices, including infant massage, sensory-motor, and cognitive stimulation.

#### Patient and public involvement

Parents and the public were not involved in study design, but participated in ensuring follow-up visits through self-reporting to the CDC.

#### Rapid Neurodevelopmental Assessment (RNDA)

A detailed RNDA form for children under age 2 years was used for data collection. Details of the development and validation of the methods used for RNDA have been published previously [12, 60]. RNDA included history of feeding practices and seizures and detailed physical and neurological examinations. General, fundoscopic, and primitive reflex examinations were conducted and levels of alertness, activity, responsiveness, near visual acuity, hearing, speech, behavior, and cognitive functions were assessed. Grading of low, moderate, or high risk for each developmental domain was made at the first visit, as described previously [12, 60]. At each subsequent visit, NDIs were identified and graded according to guidelines provided by the International Classification of Functioning [61] as mild, moderate, or severe if functions were >50%, 25 to 50%, or <25% of the gold standard, respectively [12].

#### Psychological assessment

The Bayley Scales of Infant Development (BSID II) [62] was adapted for Bangladesh [63] and was administered by a developmental psychologist at each follow-up visit for psychological assessment. Older children were administered the nonverbal section of the Stanford-Binet Intelligence Scales [64], which also was adapted for Bangladesh [65]. For a few visually impaired children (*n*=4), the Reynell-Zinkin Scales [66] was administered and the final scores were compared with those standardized for visually impaired children. Severity of cognitive impairments was graded according to the Mental Development Index (MDI) and Psychomotor Development Index (PDI) scores of the BSID II, using the following cutoff points: (1) >85 was considered as normal, (2) 71 to 85 was considered as mild impairment, and (3) <70 was considered as serious impairment. The Behavior Rating Scale results of the BSID II, graded as normal, mild, and serious, were read as low, moderate, or high risk, respectively, for the first assessment, as described previously [48]. Assessment after three months of age as (1) “within normal limits” was considered to be normal, (2) “questionable” was considered mild impairment, and (3) “suboptimal” was considered as serious impairment. Details of the methods used for psychological assessments have been described previously [12]. RNDA and psychological assessment outcomes were dichotomised into disability versus no disability.

### Statistical analysis

Data were entered into SPSS-PC (Version 11 for Windows; SPSS Inc., Chicago, Illinois) statistical software and then Stat Transfer was used to convert SPSS files into Stata files. The data were analyzed using Stata 9.2 (Stata Corp., College Station, Texas) statistical software. The outcomes of interest were neurodevelopment disabilities including gross motor, fine motor, vision, hearing, speech, cognitive, and behavioral disabilities. Longitudinal data for the outcome measures were originally collected in a scale format ranging from 1 to 4, with 1 = no NDI or disability, 2 = mild, 3 = moderate, and 4 = severe NDI or disability, as described previously [60]. Scores were dichotomized into NDI/disability (score = 2, 3, or 4) vs. no NDI/disability (score = 1) due to the limited sample size. A composite variable was created to examine any disability vs. no disability.

Socio-demographic and control variables were compared across the no treatment, SSO treatment, and Aquaphor treatment groups; sample sizes are shown for each variable and each analysis. Chi-square testing was used for categorical variables, and analysis of variance was used for continuous variables. Exploratory analysis was conducted using cross tabulation and chi-square testing. Additionally, histograms and scatter plots were used to view distributions of socio-demographic and control variables. Lowess graphs examined the relationship between the neurodevelopmental disability outcomes and age, by treatment group [67]. We used Generalized Estimating Equations for longitudinal data (xtgee command in Stata) to account for repeated measures on the same child over time, with a binomial family, logit link, exchangeable correlation and robust variance, adjusted for child age and maternal literacy [68]. Children were assumed to be independent of each other. The main effect in these models is the interaction term between treatment groups and age, which was necessary to compare treatment groups across age points.

## Results

### Subjects

Of the 216 infants who survived to discharge from the parent study, 159 infants were enrolled in the neurodevelopmental follow-up study. Some infants lived in regions and circumstances of Bangladesh which were infeasible for follow-up. Infants treated with SSO or Aquaphor were similar to control infants in family socio-demographic characteristics and in antecedent risk factors for neurodevelopmental sequelae, including maternal antenatal illness, infant gestational age and anthropometric measures, and conditions including birth asphyxia and seizures (Table 1). There were no statistically significantly differences between study group characteristics except for rural residence.

**Table 1:** Baseline characteristics of very preterm infants by treatment group (*n*=123) followed for neurodevelopmental outcomes in Bangladesh

Categorical variables	Number (***n***) (control; Aquaphor; SSO)	Control (***n***=38)	Aquaphor (***n***=45)	Sunflower seed oil (***n***=40)	***p*** value
% female: % [*n*, standard deviation (SD)]	38; 45; 35	42.1% (16)	51.1% (23)	34.3% (12)	0.317
% Rural: % (*n*, SD)	38; 45; 34	36.8% (14)	51.1% (23)	14.7% (5)	0.004
% Low income (<2000 Taka/month): % (*n*, SD)	37; 45; 34	16.2% (6)	22.2% (10)	11.4% (4)	0.531
Maternal literacy: % (*n*, SD)	37; 45; 35	83.8% (31)	82.2% (37)	82.4% (28)	0.980
Paternal literacy: % (*n*, SD)	37; 45; 34	78.4% (29)	82.2% (37)	85.3% (29)	0.749
Antenatal history of illness: % (*n*, SD)	32; 38; 31	50.0% (16)	55.3% (21)	67.7% (21)	0.342
Parental consanguinity: % (*n*, SD)	37; 42; 33	10.8% (4)	14.3% (6)	6.1% (2)	0.520
Birth asphyxia: % (*n*, SD)	36; 43; 39				
Yes		25.0% (9)	16.3% (7)	23.1% (9)	
Suspected		16.7% (6)	14.0% (6)	10.3% (4)	0.775
Neonatal seizures: % (*n*)	37; 42; 32	2.7% (1)	4.8% (2)	0.0% (0)	0.457
Mean maternal age (years): mean (SD)	36; 43; 38	24.14 (4.35)	24.60 (4.96)	24.32 (4.54)	0.903å
Mean gestational age (week): mean (SD)	38; 44: 40	31.22 (1.42)	31.06 (1.52)	31.48 (1.09)	0.374
Mean birthweight (gram): mean (SD)	38; 45; 35	1318.19 (223.68)	1291.66 (230.11)	1370.03 (245.28)	0.302
Mean birth length (centimeter, cm): mean (SD)	36; 44; 40	38.06 (2.89)	37.94 (2.91)	38.80 (2.25)	0.302
Mean birth head circumference (cm): mean (SD)	36; 44; 40	26.96 (5.01)	27.34 (1.70)	27.89 (1.42)	0.407
Mean birth mid-upper-arm circumference (cm): mean (SD)	36; 44; 40	6.86 (1.38)	6.76 (1.03)	7.14 (0.78)	0.264

### Follow-up

Of the 159 infants enrolled in the follow-up study, 26 infants died and 30 infants were lost to follow-up; 77.4% (*n*=123) attended at least one follow-up visit and were included in the study analysis, 60.4% (*n*=96) attended two or more follow-up visits, and 59.7% (*n*=95) attended follow-up visits through 30 months of age. On average, children were seen 3.7 times during the follow-up period.

#### Rapid neurodevelopmental assessment

**Fig. 1:**
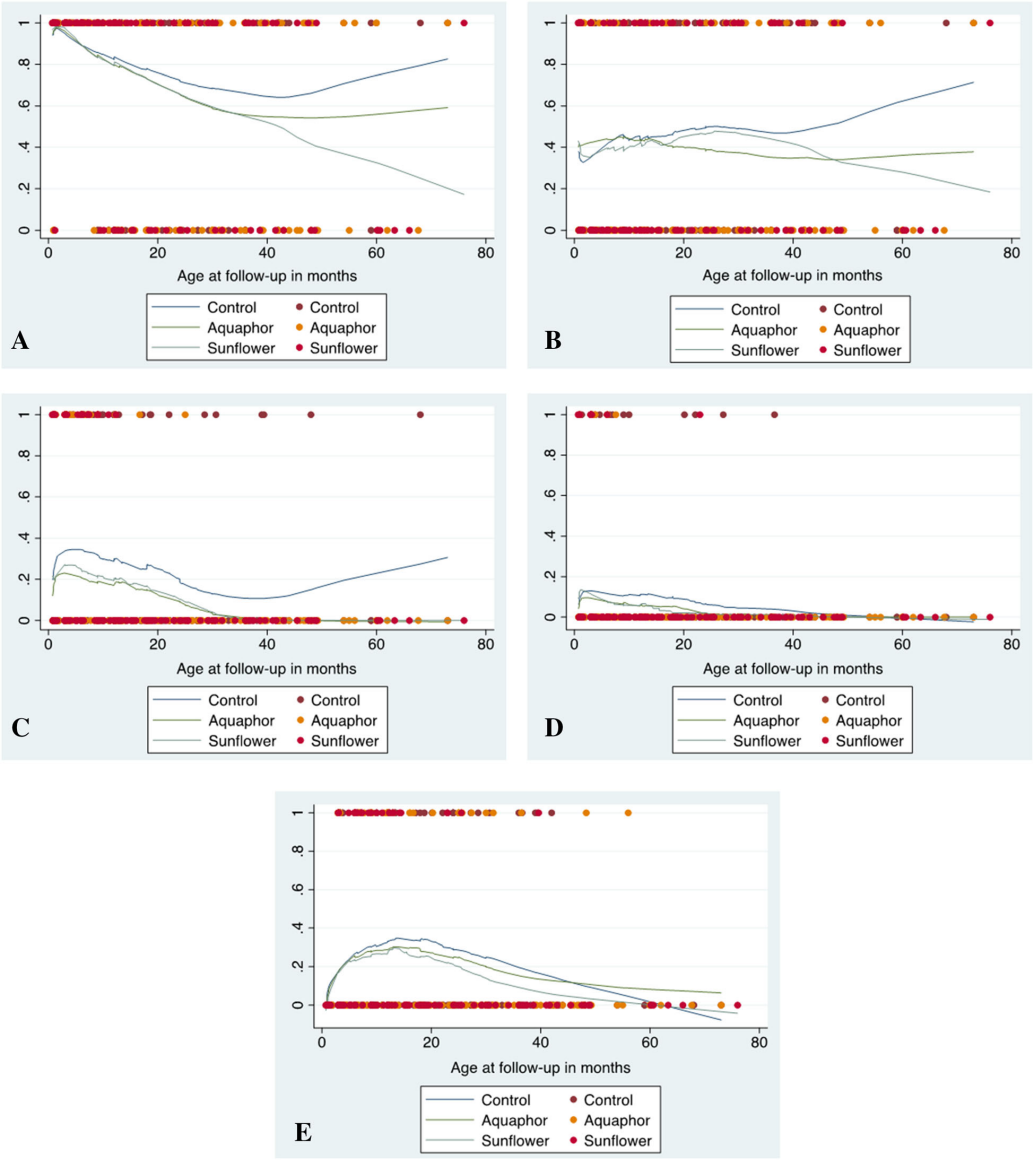
Lowess graphs of disability probability for preterm infants followed-up during early childhood in Bangladesh. **a** Probability of any disability. **b** Probability of cognitive disability. **c** Probability of fine motor disability. **d** Probability of hearing disability. **e** Probability of speech disability

Exploratory analyses suggested that there were several neurodevelopmental outcomes in which treatment groups had lower proportions of developmental delays than the control group, as seen in Lowess graphs for the composite outcome of any disability (Fig. 1a), cognitiion (Fig. 1b), fine motor skills (Fig. 1c), hearing (Fig. 1d), and speech (Fig. 1e). GEE analysis indicated that SSO had a significant protective effect on risk of fine motor skill delays (OR 0.92, 95% CI 0.86–0.98, *p*=0.006). Additionally, children in the Aquaphor group had reduced odds of having a hearing disability compared to the control group (OR 0.91, 95% CI 0.85–0.97, *p*=0.030).

#### Psychological assessment

MDI and PDI scores of the BSID II were comparable at the first assessment at 1 month of follow-up for the treatment groups and the control group (*n*=121) (Table 2). At up to 30 months of follow-up, 30.5% (29/95) and 36.8% (35/95) of children had disability on the MDI and PDI scales, respectively (Table 3). MDI scores were comparable between the control group and each of the treatment groups (Aquaphor vs. control: OR 0.80, 95% CI 0.23–2.77, *p*=0.689; SSO vs control: OR 1.16, 95% CI 0.35–3.93, *p*=0.781). The disability rate based on PDI scores was significantly lower in the Aquaphor group compared to the control group (OR 0.21, 95% CI 0.06–0.72, *p*=0.004). While the point estimate suggests protection, the SSO group was not significantly different from the control group (OR 0.49, 95% CI 0.15–1.52, *p*=0.167) (Table 3). Behavior Rating Scales of the BSID revealed ‘questionable’ scores (i.e., mild impairment) which were comparable across all three groups at both the one-month assessment and the assessment at 30 months (data not shown).

**Table 2:** Psychological assessment using Bayley Scales of Infant Development II at baseline (one month of age)

Score	Control (*n*=39)	Aquaphor (*n*=43)	Sunflower seed oil (*n*=39)	Total (*n*=121)	*p* value
**Mental Development Index**					
>85	26 (66.7%)	23 (53.5%)	21 (53.9%)	70 (57.9%)	0.4533
>70-85	13 (33.3%)	17 (39.5%)	16 (41.0%)	46 (38.0%)	
<70	0 (0.0%)	3 (7.0%)	2 (5.1%)	5 (4.1%)	
**Psychomotor Development Index**					
>85	17 (43.6%)	17 (39.5%)	14 (35.9%)	48 (39.7%)	0.3292
>70–85	17 (43.6%)	19 (44.2%)	13 (33.3%)	49 (40.5%)	
<70	5 (12.8%)	7 (16.3%)	12 (30.8%)	24 (19.8%)

**Table 3:** Psychological assessment using Bayley Scales of Infant Development II at 30 months of age

Category	Control (*n*=29)	Aquaphor (*n*=34)	OR (95% CI) Aquaphor vs. control	*p* value	Sunflower seed oil (SSO) (*n*=32)	OR (95% CI) SSO vs. control	*p* value	Total (*n*=95)
**Mental Development Index**								
No disability^a^	20 (69.0%)	25 (73.5%)			21 (65.6%)			66 (68.5%)
Disability^b^	9 (31.0%)	9 (26.5%)	0.80 (0.23–2.77)	0.689	11 (34.4%)	1.16 (0.35–3.93)	0.781	29 (30.5%)
**Psychomotor Development Index**								
No disability^a^	13 (44.8%)	27 (79.4%)			20 (62.5%)			60 (63.2%)
Disability^b^	16 (55.2%)	7 (23.6%)	0.21 (0.06–0.72)	0.004	12 (37.5%)	0.49 (0.15–1.52)	0.167	35 (36.8%)

^a^Score >85

^b^Score <85

## Discussion

These findings build on the parent emollient study that demonstrated benefits of emollient therapy for improved skin barrier function, and prevention of serious infections and mortality of very preterm infants during the neonatal period [47–49, 55]. Here, we show that treatment with SSO reduced the risk for development of disability in fine motor skills and Aquaphor reduced the risk of hearing disability, perhaps due to protection from infection. Early acquisition of fine motor skills is important for school readiness and is predictive of later educational achievement, for example in math reading and science [69, 70]. Infants in the Aquaphor group also had significantly improved psychomotor development. Developmental therapists provided education to mothers in all three groups on how to perform massage at home during the follow-up period, which may have attenuated differences between groups.

Previous investigations have shown benefits of massage for preterm infants [29], with improved scores on several clusters on the Brazelton Neonatal Behavior Assessment Scales, including habituation, motor skills, range of state, autonomic stability, excitability, stress behaviors, and more time in active alertness [37]. Better performance on the Brazelton Scales may have facilitated early parent-infant interactions, which in turn, may have benefitted the subsequent development of these preterm infants. In an 8-month follow-up study of preterm infants randomized to massage or no massage while in the Neonatal Intensive Care Unit, massaged infants performed better on the Bayley Mental and Motor Scales [25]. Another study found superior performance on the Bayley Mental Scale later in the first year in preterm infants who received massage interventions, in addition to superior habituation performance noted at the end of the neonatal period [71]. Higher cognitive scores at 12 months of corrected age were also found in infants who received massage, while weight and motor scores did not differ between groups [72].

Limited evidence suggests that benefits of emollient therapy extend beyond those due to massage alone. One study comparing massage with SSO to massage without oil and to no massage showed that weight gain in the SSO massage group was higher compared to the massage only and the no massage groups [37, 51], suggesting that absorption and metabolism of the oil contributed to weight gain [50, 52, 53]. However, there was little statistical difference in the Brazelton Neonatal Behavior Assessment Scales post intervention [37].

SSO is a low-cost, widely available emollient in low-income countries. Topical emollient therapy is a common-to-universal newborn infant and child care practice throughout many low-resource countries [41–45]. A course of SSO treatment of a newborn weighing 1.5 kg costs $0.20 [47]. The higher cost and lack of consistent availability in the marketplace of Aquaphor may prove difficult in low income settings. Moreover, the cost-effectiveness of SSO was superior to Aquaphor in the parent trial to this study [47], which showed that SSO cost US$ 61 per death averted and US$ 2.15 per YLL averted (I$ 6.39, international dollars, per YLL averted) while Aquaphor cost US$ 162 per death averted and US$ 5.74 per YLL averted (I$ 17.09 per YLL averted) [56].

There were several limitations to this study. Our study enrollment and stratification were based on estimates of gestational age—the average of three separate measures—yet we lacked gold-standard ultrasound-based estimates of gestational age. The sample size became prohibitively small as participants were stratified by disability type, age, and treatment groups and were lost to follow-up primarily due to long distances from subjects’ homes to Dhaka Shishu Hospital. This analysis is best viewed as a hypothesis-generating exercise requiring further study in understanding benefits of emollient therapy on long-term neurodevelopment. Additionally, this study was unable to distinguish between the effects of massage and emollients because there was no massage only group. This was deliberate to protect the preterm infants’ skin integrity from the friction of massage without oil.

## Conclusion

In conclusion, results show that emollient therapy of preterm infants with SSO or Aquaphor during the neonatal period shows promise for improving child neurodevelopmental outcomes. Resource and income-limited populations may benefit from incorporating improved emollient practices for improved long-term neurodevelopment along with (as shown in prior analyses) improved neonatal weight gain and reduced risk for serious infections and mortality. Further research into impacts and mechanisms of emollient therapy in improving child neurodevelopment is recommended.

## Data Availability

Data are available upon reasonable request to the corresponding author, through a data sharing agreement with Stanford University.

## Abbreviations

BSID: Bayley Scales of Infant Development
cm: Centimeter
CDC: Child Development Centre
CHWs: Community health workers
CI: Confidence interval
GEE: Generalized Estimating Equations
LMICs: Low- and middle-income countries
MD: Mean difference
MDI: Mental Development Index
NDIs: Neurodevelopmental impairments
*n*: Number
RNDA: Rapid Neurodevelopmental Assessment
OR: Odds ratio
PDI: Psychomotor Development Index
RR: Relative risk
SD: Standard deviation
STROBE: Strengthening the Reporting of Observational Studies in Epidemiology
SSO: Sunflower seed oil
